# Celiac Disease and Liver Disorders: From Putative Pathogenesis to Clinical Implications

**DOI:** 10.3390/nu10070892

**Published:** 2018-07-12

**Authors:** Iva Hoffmanová, Daniel Sánchez, Ludmila Tučková, Helena Tlaskalová-Hogenová

**Affiliations:** 1 Centre for Research on Nutrition, Metabolism and Diabetes, Third Faculty of Medicine, Charles University, Ruska 87, 100 00 Prague, Czech Republic; 2 Second Department of Internal Medicine, Third Faculty of Medicine, Charles University, Ruska 87, 100 00 Prague, Czech Republic; 3 Laboratory of Cellular and Molecular Immunology, Institute of Microbiology of the Czech Academy of Sciences, v.v.i., Videnska 1083, 142 20 Prague, Czech Republic; sanchez@biomed.cas.cz (D.S.); ltuckova@gmail.com (L.T.); tlaskalo@biomed.cas.cz (H.T.-H.)

**Keywords:** autoimmunity, celiac hepatitis, gut–liver axis, liver immunity, non-alcoholic fatty liver disease, tolerance, intestinal barrier

## Abstract

Immunologically mediated liver diseases belong to the common extraintestinal manifestations of celiac disease. We have reviewed the current literature that addresses the association between celiac disease and liver disorders. We searched relevant articles on MEDLINE/PubMed up to 15 June 2018. The objective of the article is to provide a comprehensive and up-to-date review on the latest hypotheses explaining the pathogenetic relationship between celiac disease and liver injury. Besides the involvement of gut–liver axis, tissue transglutaminase antibodies, and impairment of intestinal barrier, we integrate the latest achievements made in elucidation of the role of gut microbiota in celiac disease and liver disorders, that has not yet been sufficiently discussed in the literature in this context. The further objective is to provide a complete clinical overview on the types of liver diseases frequently found in celiac disease. In conclusion, the review highlights the clinical implication, recommend a rational approach for managing elevated transaminases in celiac patients, and underscore the importance of screening for celiac disease in patients with associated liver disease.

## 1. Introduction

The liver is an organ playing a regulatory role in innate and adaptive immunity. Constituents in portal blood circulating from the intestine to the liver, such as food antigens (including peptides derived from gluten and related cereals), bacterial components and products, can under specific conditions (e.g., impaired intestinal barrier and/or dysregulation of gut–liver axis) trigger the hepatic immune response, which is connected to liver inflammation and fibrosis. 

During the past four decades a vast number of papers documented the clinical relationship between celiac disease and liver disorders. Although immunologically mediated liver diseases are the most common extraintestinal manifestations of celiac disease [1,2,3,4,5,6,7,8], there are still many scientific and clinical question to be answered. 

This narrative review attempts to summarize the current status of knowledge regarding (1) the pathogenetic relationship between celiac disease and liver injury, (2) the types of liver diseases frequently found in celiac disease, (3) recommendation for a rational approach for managing elevated transaminases in celiac patients, and (4) *vice versa* for screening for celiac disease in patients with liver diseases. A search for English written articles was performed using MEDLINE/PubMed (from 1977 up to 15 June 2018). We included scientific papers regarding liver immunology to emphasize liver as an organ with unique immune function, and to summarize hypotheses explaining connection between celiac diseases and liver disorders. Especially, we highlight the gut microbiota as further possible link clarifying the relationship between liver injury and celiac disease. In addition, number of observational studies and meta-analyses were used to document the current clinical approach to the liver disorders in celiac patients. 

## 2. Immune Reactivity in the Liver

The liver, traditionally viewed as the central organ engaged in metabolism, nutrient storage, and detoxification, is also an immune organ, having an important regulatory role in innate and adaptive immunity. From an immunological point of view, the liver is exposed to large numbers of foreign molecules coming from the gastrointestinal tract via the portal vein. These non-self-antigens are derived from food (including peptides derived from gluten and related cereals), and from the microbiota (i.e., bacterial components and products) that have breached the intestinal barrier. The liver must tolerate these gut–derived molecules, while still providing immunosurveillance for pathogenic infections and malignant cells. The liver sinusoids and the sub-endothelial compartment (the space of Disse) are populated with various resident immune cells (of the innate and adaptive immune system) capable of dynamic interaction, antigen sensing, phagocytosis, antigen presentation, cytokine and chemokine production, cytotoxicity, T cells programing, and communication with circulating immune cells passing through the liver. The diversity of immune interplay determines the balance between tolerance, protective immunity against infection, tissue damage, and metastases, and autoimmunity in the liver. Variety of antigen-presenting cells (APCs), such as liver-resident macrophages, called Kupffer cells, liver-resident dendritic cells, and liver sinusoidal endothelial cells, play a central role in creating the tolerogenic environment of the liver. Kupffer cells act as the primary filter, constantly removing antigens from the circulation. The liver is particularly enriched with populations of resident innate lymphoid cells (natural killer (NK) cells, natural killer T (NKT) cells, mucosal-associated invariant T cells, and γδ T cells), which recognize conserved antigens (expressed by microbial pathogens, pathogen-infected cells, and tumor cells), and respond by killing target cells, or selectively activating and regulating the diverse arms of both the innate and adaptive immune responses in the liver, including T helper (Th)1, (Th)2, (Th)17, T_reg_ cells, and antibody responses. Lymphoid cells of adaptive immunity comprise the classic major histocompatibility complex (MHC)-restricted CD4+ and CD8+ T cells, activated T cells, memory T cells, as well as B cells. Besides the above mentioned professional APCs, parenchymal cells and hepatic stellate cells act as semi-professional APCs, which can work as sensors or triggers of immune responses and assist in local or systemic immune regulation and inflammation. All types of liver cells, including resident immune cells, hepatocytes, cholangiocytes, sinusoidal endothelial cells, and hepatic stellate cells express a range of pattern recognition receptors (PRRs), such as toll-like receptors (TLRs), NOD-like receptors (NLRs), RIG-like receptors, carbohydrate receptors, and scavenger receptors, which bind to microbial-associated molecular patterns (MAMPs) and damage-associated molecular patterns (DAMPs) that may be present in portal blood. Kupffer cells, liver sinusoidal endothelial cells, and hepatocytes also express variable numbers of class II MHC molecules and are capable of presenting antigens to classic T cells [9,10]. 

The liver is a highly immunotolerant organ with many mechanisms to actively prevent the induction of immunity. While maintaining an overall tolerogenic setting, the hepatic immune system must be capable of rapidly activating immune responses to “dangerous” antigens or to liver tissue damage (of any origin). Immune tolerance to self-antigens in the liver can be impaired, which can lead to autoimmune liver diseases, such as autoimmune hepatitis (AIH), primary biliary cholangitis (PBC), and primary sclerosing cholangitis (PSC). The hepatic immune system plays an important role in responding to hepatic injury, and in the initiation and progression of liver diseases. Thus, chronic liver diseases of infectious, toxic, metabolic, cholestatic, and autoimmune origin are characterized by persistent activation of innate immune pathways, which are usually associated with cytopathic effects on liver parenchymal cells [9,10].

About 1–2% of food antigens are translocated into the circulation in an immunogenic form [11]. The liver is able to suppress immunity to many antigens, including dietary antigens. Additionally, various dietary constituents and metabolites can influence hepatic APCs and other liver cells (mainly by activating specific receptors, leading to altered intracellular signaling, gene expression, and release of cytokines) and therefore modulate physiological immune maintenance in the liver. Dietary constituents and metabolites can also increase pathological activation of liver immune cells. Liver metabolic processes are linked to hepatic inflammation through the inflammatory effects of metabolites such as saturated fatty acids, cholesterol, or succinate, which promote TLRs signaling and inflammasome activation on dendritic cells and macrophages [9,10]. Data sets published on the potential influence of dietary components, such as branch-chain amino acids, arginine, tryptophan, vitamins, and various types of lipids, on hepatic immunity show equivocal or conflicting results in terms of whether they induce tolerance or initiate an immune response [10]. Similarly, the role of dietary gluten and other immunogenic wheat proteins, in the modulation of hepatic immunity, has not been elucidated. 

## 3. Gut–Liver Axis and Celiac Disease

**Figure 1:**
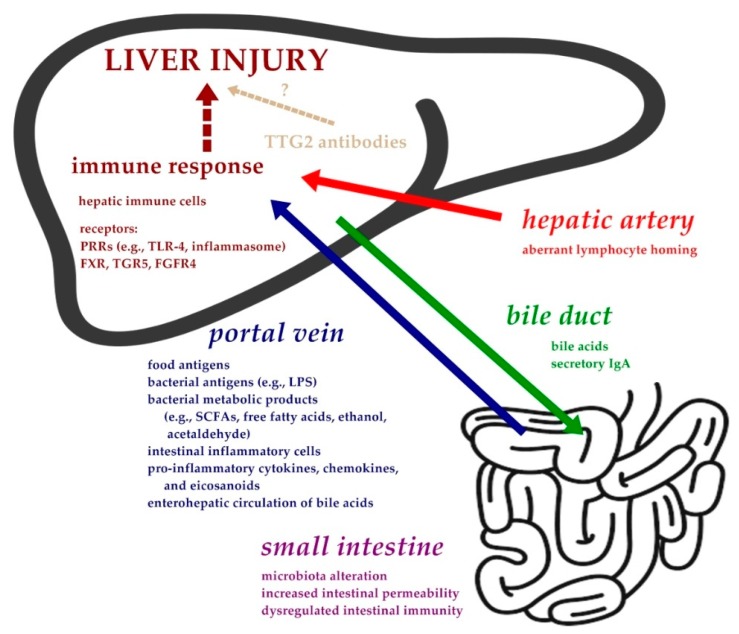
Putative pathogenesis of liver injury in celiac disease. Abbreviations: FGFR4, fibroblast growth factor receptor 4; FXR, farnesoid X receptor; LPS, lipopolysacharide; NLRs, NOD like receptors; PRRs, pattern-recognition receptors; SCFAs, short chain fatty acids; TGR5, Takeda G-protein receptor 5; TLR-4, toll like receptor 4; TTG2, tissue transglutaminase 2.

Although the relationship between celiac disease (CD) and liver disorders have been clinically evident for more than 40 years [1], its pathophysiology is far from being fully understood with many explanations being hypothetical. In addition to a probable genetic relationship, the interplay of the many aspects of the gut–liver axis may also contribute to the explanation of this link (Figure 1). 

The term gut–liver axis describes a close anatomical, metabolic, and immunologic connection between the gut and liver. The liver and intestine are tightly linked via the portal circulation, which supplies the liver not only with nutrients but also with gut–derived food and bacterial antigens, and bacterial metabolic products. The liver portal and liver arterial circulation are the afferent part of the gut–liver axis, while the biliary tree is the efferent part of the gut–liver axis. Intestinal and biliary epithelia share many properties, including expression of PRRs and tight junction (TJs) proteins, as well as the ability to release secretory IgA. The secretion of bile (especially bile acids, and IgA) affects the gut–liver axis and modulates the composition of the intestinal microbiota [12]. Moreover, both organs are characterized by shared lymphocyte homing and recruitment pathways. Gut-derived T-lymphocytes may also contribute to hepato-biliary inflammation [13].

The gut–liver axis is a complex system involving multiple components—the intestinal barrier, gut microbiota, bile, shared lymphocyte homing, and several hepatic receptors, such as PRRs, or farnesoid X receptor (FXR), Takeda G-protein receptor 5 (TGR5), fibroblast growth factor receptor 4 (FGFR4)—that connect metabolic pathways to inflammation. Dysregulation or impairment of the gut–liver axis can activate the hepatic innate immune response leading to induction of liver injury, or contribute to the progression of liver damage (of any origin) [12,14] (Table 1).

When the intestinal barrier is impaired (i.e., during increased intestinal permeability or dysregulated intestinal immunity), food antigens and bacterial antigens with strong immune-activating properties (e.g., lipopolysaccharide (LPS), peptidoglycans, super-antigens, bacterial DNA, flagellin, and heat shock proteins) can cross the intestinal epithelium in greater numbers than under healthy conditions, which can stimulate gut–associated lymphatic tissue (GALT) to release pro-inflammatory cytokines (TNF, IL-1, IL-6, etc.), chemokines, and eicosanoids. The load of antigens is not eliminated by GALT and together with intestinal inflammatory cells, cytokines, chemokines, and bacterial metabolites (such as ethanol, acetaldehyde, trimethylamine, short chain fatty acids (SCFAs), and free fatty acids) are transported to the liver via the portal vein. In the liver, the massive influx of these components activates the hepatic immune response thus promoting liver damage, inflammation, and fibrogenesis. Increased intestinal permeability as well as plasma levels of inflammatory cytokines has been demonstrated in both the pathophysiology and progression of chronic liver diseases. Similarly, increased levels of bacterial LPS have been documented in the portal and/or systemic circulation (endotoxemia) in several chronic liver diseases [12,13,15,16].

Among the compounds that can disrupt the function of the intestinal barrier are known dietary items or eating habits (e.g., alcohol use, high-fat, or high-carbohydrate diets), intestinal mucosal inflammation (regardless of etiology), infections, toxins, medications (e.g., non-steroidal antirheumatic drugs, proton-pump-inhibitors), and intestinal hypoperfusion [12]. It is also hypothesized that industrial food additives (such as gluten, microbial transglutaminase, glucose, salt, emulsifiers, organic solvents) that are commonly used in dough, may have a negative impact on the function of the intestinal barrier [17]. Another leading factor influencing intestinal permeability is the microbiota [18].

In CD, increased intestinal permeability is thought to be an early feature in the pathogenesis. Dietary gluten drives inflammation and impairs the function of the small intestinal barrier [19,20]. Using markers of epithelial apoptosis (cytokeratin 18 caspase-cleaved fragment) and enterocyte damage (intestinal fatty acid-binding protein), we found an impairment of small intestinal mucosal barrier integrity in patients with active celiac disease and also in patients with diabetes mellitus [21]. It has been demonstrated that patients having active CD with elevated serum transaminases have a more severely compromised intestinal barrier and greater intestinal permeability than those having active CD with normal liver tests; and both intestinal permeability and transaminases normalize when patients follow a gluten-free diet (GFD) [20,22]. 

In CD, gliadin *per se* has been shown to increase intestinal permeability by releasing zonulin—a modulator of small-intestinal TJs [13,23]. Luminal gliadin binds to the chemokine receptor, CXCR3, expressed on intestinal epithelium, and induces an MyD88-dependent zonulin release, which leads to an opening of enterocyte TJs. CXCR3 is over-expressed in CD patients, where it is co-localized with gliadin. CXCR3 is predominantly expressed on immune cells, but its expression has also been reported on non-immune cells, including hepatic parenchymal cells. Resident hepatic immune cells and hepatocytes express CXCR3 in both the normal and injured liver. While the role of these receptors in the homeostatic liver environment is not understood, during injury, they are engaged in cellular survival, activation, proliferation, apoptosis, inflammatory cell infiltration, fibrogenesis, angiogenesis, and expression of additional chemokines and growth factors [24]. Up-regulated production of CXCR3 ligands under inflammatory gut–liver interactions was recently demonstrated using an integrative multi-organ platform comprising human liver (hepatocytes and Kupffer cells) and intestinal (enterocytes, goblet cells, and dendritic cells) models [25]. 

It is not known, if gluten can affect intestinal permeability and hepatic immunity in healthy individuals. But, studies in murine monocytes/macrophages have demonstrated that gluten peptides activate the arginase metabolic pathway and increase intestinal permeability in vitro (on Caco-2 epithelial monolayers) [26]. Gliadin activated monocytes/macrophages contribute to the innate immune response in CD [27] by releasing inflammatory cytokines and nitric oxide (NO). Arginine is an obligatory substrate of inducible NO synthase, an enzyme that produces large amounts of NO. Moreover, it has been recently demonstrated that in both celiac subjects and healthy individuals, gluten peptides exert the same level of activation of arginine metabolism and cell polarization in human monocytes; therefore, ingested gluten might elicit innate activation of human monocytes even in healthy people [28]. 

## 4. Involvement of Microbiota in Celiac Disease and Liver Disorders

Dysbiosis (i.e., a reduction in bacterial diversity of beneficial bacterial species, and an increase in potentially pathogenic species relative to a healthy microbiome) is a feature of CD as well as both early and advanced liver disease. 

CD is strongly associated with intestinal dysbiosis that is not completely restored by a GFD. Although, no distinct celiac microbiota pattern has yet to be found, an altered composition of fecal and duodenal microbiota has been identified in individuals with active celiac disease: e.g., an abundance of Firmicutes and Proteobacteria, or of Gram-negative bacteria (such as *Bacteroides* and *E. coli*), or *Staphylococcus* and *Clostridium*. This is accompanied by a related reduction in protective, anti-inflammatory bacteria (such as *Bifidobacterium* and Lactobacillus), and Gram-positive bacteria [19,29]. Microbiota might contribute to the pathogenesis and manifestation of CD via proteolytic activity that can modify the immunogenicity of gliadin peptides or by influencing intestinal permeability and inflammation [30]. 

There is also growing evidence that perturbations in the microbiota composition (dysbiosis or bacterial overgrowth) contribute to the pathogenesis (manifestation and/or maintenance and progression) of certain liver diseases, such as non-alcoholic fatty liver disease (NAFLD) (and its progressive variant, non-alcoholic steatohepatitis (NASH)), alcohol-related liver disease, primary sclerosing cholangitis (PSC), primary biliary cholangitis (PBC), and even liver cirrhosis and its complications including hepatocellular carcinoma [31,32]. The mechanism by which the microbiota affects intestinal permeability and TJs function is unknown. Several studies have shown that dysbiosis contributes to intestinal inflammation, bacterial translocation, and liver fibrosis [33].

The MAMPs are recognized by PRRs, such as TLRs or NLRs, which are expressed on intestinal epithelial and antigen presenting cells, and on liver resident immune and parenchymal cells. The CD14/TLR-4 complex (which is involved in the recognition and signal transduction of bacterial LPS) plays a central role in the initiation or maintenance of innate immune responses in the small intestine and in the liver and contributes to impairment of intestinal barrier [30]. 

In active celiac disease, increased TLR4 (and TLR2) expression in the duodenum has been documented [34]. In addition, our group reported increased sCD14 protein seropositivity in active CD. Soluble CD14 (sCD14) is considered to be an indicator of innate immunity activation in response to mucosal translocation of LPS. So, in CD, circulating LPS might contribute to innate immune activation of extraintestinal organs, including the liver [21]. 

In experimental models, the evaluation of specific bacteria isolated from celiac patients suggests that some commensal bacteria might promote an adverse response to dietary gluten, whereas others can be protective [35], and that gut microbiota can enhance or reduce celiac-disease-associated immunopathology (such as modifying T_reg_ induction, epithelial cell stress, and activation of intraepithelial lymphocytes, maturation of dendritic cells, pro-inflammatory cytokine production, intestinal permeability, and the induction of CD4+ T cell responses) [29]. Gut microbiota promotes chronic liver inflammation and fibrogenesis through the activation of TLR4 (on Kupffer cells and hepatic stellate cells). It has been demonstrated in mice, that bacterial LPS enhance TGF-β signaling in hepatic stellate cells, via the TLR4-MyD88-NF-κB pathway, subsequently leading to liver fibrogenesis [36]. Intestinal dysbiosis is associated with increased levels of LPS in the portal and/or systemic blood (endotoxemia) and propagates liver injury in several types of chronic liver diseases [12]. 

In addition to TLR4, inflammasome activation contributes to the pathogenesis of most acute and chronic liver disease. Inflammasomes are multiprotein complexes (involving NLRs) that can recognize a diverse range of MAMPs and DAMPs. Inflammasome-mediated dysbiosis enhances liver inflammation through activation of caspase-1 cascade and production of various proinflammatory cytokines (such as IL-1, IL-18, IL-6, TNF-α), enhances hepatocyte dysfunction, necrosis, apoptosis, and the generation of extracellular fibrosis [37]. 

Moreover, the activation of TLRs and inflammasome in hepatocytes seems to also be involved in metabolic regulation, since dysbiosis may alter nutritional absorption and storage [38].

Dysbiosis may also influence bile acid signatures in the gut and in enterohepatic circulation. Gut microbial enzymes (bile salt hydrolase, or bile acid-inducible enzymes) deconjugate and dehydroxylate primary bile acids in the intestine into unconjugated secondary bile acids. Bile acids (besides their well-known role in nutrient absorption and biliary secretion of lipids, toxic metabolites, and xenobiotics) are ligands for liver bile salt receptors, such as the nuclear FXR and membrane TGR5 [14]. Hepatic FXR plays a key role not only in regulation of hepatic bile acids metabolism, but also in the coordination of hepatic triglyceride, glucose, and energy homeostasis [39]. TGR5 is localized on various non-parenchymal liver cells (sinusoidal endothelial cells, Kupffer cells, hepatic stellate cells) and in cholangiocytes. Activation of TGR5 mediates choleretic, cell-protective, anti-inflammatory, and proliferative effects in cholangiocytes. A disturbance in the bile acids signaling mechanisms can contribute to the development or progression of biliary diseases, such as primary biliary cholangitis (PBC) and primary sclerosing cholangitis (PSC) [40]. Moreover, bile acids also influence hepatic inflammation and regeneration (via the intestinal bile acids-intestinal FXR-fibroblast growth factor (FGF) 15/19 and liver FGF receptor 4 axis). The alteration in the bile acid profile might provide signals that connect bile acid, lipid, and glucose metabolism in the liver to hepatic and biliary inflammation. Bile acids also have antimicrobial properties and therefore can modulate gut microbiota composition, intestinal immunity, and barrier integrity [14]. 

## 5. The Role of Tissue Tranglutaminase Antibodies and Vitamin D in a Pathogenetic Link between Celiac Disease and Hepatic Disorders

Beside the contribution of the gut–liver axis to hepatic injury in CD, it has also been suggested that tissue transglutaminase 2 (TTG2) (a key enzyme and part of target autoantigen involved in the pathogenesis of celiac disease [19]) may play a role in celiac-associated liver damage. This hypothesis is supported by observation that chronic unexplained hypertransaminasemia is frequently present in active CD, but not in other small intestinal diseases associated with dysbiosis and an increased inflammatory mucosal response, such as tropical sprue or diarrhea predominant-irritable bowel syndrome [41]. 

TTG2 is a ubiquitous cellular and extracellular enzyme, participating in important biological processes such as wound healing, tissue repair, fibrogenesis, apoptosis, inflammation, and cell-cycle control. In the extracellular space, TTG2 is involved in cell-extracellular matrix interactions as well as in remodeling and stabilization of the extracellular matrix [42]. It is also known to modulate inflammation and fibrosis in chronic liver diseases, although the mechanism appears to be quite complex and is not completely understood [43]. TTG2 is overexpressed in the liver (mainly in endothelial cells and periportal hepatocytes) of patients suffering from chronic liver diseases. Our group documented that overexpression of TTG2 is more pronounced in the liver tissue of patients suffering simultaneously from both liver disease (such as toxic hepatitis, primary sclerosing cholangitis, or autoimmune hepatitis type I) and active CD [44].

There is also evidence that enhanced TTG2 enzymatic activity seems to protect the liver from acute and chronic injury [43]. In CD, the presence of celiac antibodies has been shown to inhibit TTG2 enzymatic activity, which can lead to a decrease in the availability of active transforming growth factor (TGF)-β [45]. IgA antibodies targeted against TTG2 (IgA-TTG2 deposits), which have been found in liver biopsy specimens from patients with active CD and elevated liver transaminase values, and also in skeletal muscle, the appendix, and lymph nodes, suggest that alteration in TTG2 bioactivity (i.e., inhibition by anti-TTG2 antibodies) might play a role in celiac extra-intestinal manifestations, including liver injury [46]. We described that also another autoantigen, calcium binding protein calreticulin, could be involved in putative pathogenesis of liver injury and celiac disease [16,47].

Moreover, vitamin D deficiency, which is commonly found in active celiac disease [48], could also contribute to a proinflammatory state in the liver. Vitamin D is known for its anti-inflammatory effects on immune cells, which might be particularly relevant to the liver’s immune response. For instance, vitamin D inhibits the inflammatory maturation of dendritic cells, influences the responsiveness of macrophages to LPS and other MAMPs, and facilitates homing of activated T cells [10].

## 6. Celiac Disease and Liver Disorders in the Clinical Context

Although CD is associated with a wide spectrum of liver disorders, two distinct forms can be distinguished: (1) cryptogenic hypertransaminasaemia (celiac hepatitis), and (2) associated autoimmune liver diseases (AILD), such as autoimmune hepatitis (AIH), autoimmune cholangitis, primary biliary cholangitis (PBC), primary sclerosing cholangitis (PSC). Cryptogenic hypertransaminasaemia (celiac hepatitis), a common extraintestinal presentation of CD, is closely related to gluten intake. Additionally, CD can simply coexist as a coincidental finding with several liver diseases, such as non-alcoholic fatty liver disease (NAFLD) (and its subgroup termed non-alcoholic steatohepatitis (NASH)), chronic viral hepatitis B or C, alcoholic liver disease, hemochromatosis, Wilson’s disease, and other hereditary hepatic diseases [3,44,49,50,51]. Swedish epidemiological studies have revealed that patients with CD have an increased risk of both prior and subsequent liver disease, four-times and six-times, respectively [52], and an eight-times increased risk of mortality from liver cirrhosis [53]; however, no increased risk of liver transplantation was found (HR, 1.07; 95% CI, 0.12–9.62; *p* = 0.954) [52]. Similarly, in the large-scale screening study, we observed that seropositivity for IgA anti-TTG antibodies in patients with various liver diseases is higher (3%) [44] then prevalence of celiac disease in the general population [54].

### 6.1. Celiac Hepatitis

Isolated elevation of serum transaminases (alanine aminotransferase (ALT) and aspartate aminotransferase (AST)) is often present in newly diagnosed, active CD. The liver injury in CD, when other concomitant hepatic disease is absent, and that completely resolves after GFD, has been termed “celiac hepatitis” [55]. The reported prevalence of elevated serum transaminase levels in active celiac diseases varies from 10–50% [2,3,4,5,6]. 

Elevated transaminases do not correlate with sex, weight, height, or body mass index [4,5,6]. Some studies found no correlation with CD symptoms at diagnosis and found that patients were usually asymptomatic [5,6,56]. While others have reported an association between hypertransaminasemia and malabsorption and diarrhea, or poor growth in children [4,6]. In a study performed by Aarela et al., factors associated with increased ALT were poor growth and severe villous atrophy. There was a moderate statistically significant correlation between ALT and endomysial antibodies (*r* = 0.334, *p* < 0.001), TTG2 antibodies (*r* = 0.264, *p* = 0.002), and ferritin (*r* = −0.225, *p* = 0.03), but not with other laboratory values [6]. Similarly, in adult celiac patients, one study found a correlation between hypertransaminasemia and malabsorption (odds ratio (OR), 2.22; *p* = 0.004), diarrhea (OR, 1.72; *p* = 0.005), and increasing severity of mucosal lesions (OR, 1.46; *p* = 0.001) [4]. By contrast, another study reported that the severity of intestinal histological changes did not correlate with transaminases levels [57]. Hypertransaminasaemia has been shown to correlate with intestinal permeability. In active CD, the intestinal permeability index (% lactulose/% mannitol in a 5 h urine collection) is statistically significantly (*p* < 0.0001) higher in patients with elevated transaminases and is correlated with AST (tau = 0.34; *p* < 0.0001) and ALT (tau = 0.32; *p* < 0.0001) [22].

Generally, hypertransaminasaemia is mild. Values greater than 5-time the ULN (upper limit of normal) are seen in a minority of patients [4,5]. The clinical presentation of celiac hepatitis is often asymptomatic but can vary up to advanced liver disease. Chronic untreated CD may also lead to chronic hepatitis and consequent liver cirrhosis, including, although seldom, end-stage liver cirrhosis requiring liver transplantation [58,59]. Acute cryptogenic liver failure, which resolved after a GFD without the necessity for liver transplantation, has been, although rarely, described in untreated CD [58,60], or CD that was diagnosed long after liver transplantation due to “cryptogenic” liver cirrhosis [61]. In childhood CD, six cases of severe liver disease (two of them needing liver transplantation) have been reported [62]. 

No histologic findings are pathognomonic for celiac hepatitis. Non-specific reactive hepatitis is the mostly described lesion, but the histologic picture varies from normal liver architecture, or minimal lymphocytic infiltrates of portal tracts, to mild or moderate reactive hepatitis, steatosis, fibrosis, and rarely to late morphological changes, such as advanced fibrosis or cirrhosis. Inflammatory alterations of the bile ducts have not been found [1,44,56,63].

Celiac hepatitis is characterized by the presence of liver injury that resolves after introduction of a GFD. In the majority patients, a GFD leads to normalization of previously elevated transaminases levels within 1 year [2,3,4,5,6,60]. This suggests that gluten intake (that is responsible for small intestinal damage and the autoimmune reaction) drives the pathogenesis of celiac hepatitis.

Only a small percentage of patients with celiac hepatitis will need longer periods for complete normalization of liver tests. The time needed probably reflects the time required for complete intestinal mucosal recovery, which has been shown to take up to 3–5 years after the initiation of a GFD [64,65].

The relationship between hypertransaminasemia and gluten intake in celiac hepatitis has also been documented in other observations: (1) liver transaminases remained elevated during GFD non-compliance [66], (2) even normal transaminase levels decreased significantly on a GFD, and (3) a gluten challenge can lead to mild but transient hypertransaminasemia [67]. Several other observations have shown histologic improvement of liver injury (i.e., a decrease in steatosis, portal and lobular inflammation, and fibrosis score) on a GFD [63]. 

### 6.2. Associated Autoimmune Liver Diseases 

The association between CD and AILD is more uncommon than celiac hepatitis, although it has been widely demonstrated. According to systematic reviews, the prevalence of CD in AIH is 6.3% in children [2] and about 4% in adults [7]. The prevalence of CD in PBC varies from 3–7%, and in PSC from 2–3% [8]. 

The pathophysiology of CD associated AILD is not driven directly by gluten, since good adherence to a GFD alone usually does not lead to an improvement in liver tests or in the course of hepatic disease [3,8,44,49,50]. Patients with AILD and CD require both specific immunosuppressive therapy for the liver disorder as well as a GFD. GFD only improves symptoms related to CD while decreasing the risk of long-term CD complications. It is unknown if the prognosis of patients with AILD associated with CD is different from patients with AILD alone. The clinical impact of a GFD on the progression of AILD, therefore, remains uncertain [68,69]. Although, studies carried out on a small number of pediatric patients with AIH showed that children with AIH and CD on a GFD achieved treatment-free sustained remission in a significantly higher proportion compared to those without CD [70,71]. Another study reported that several children with CD and AIH developed relapses during a spontaneous gluten challenge [72]. So, a GFD may prevent an early relapse of AIH in patients having AIH associated with CD. 

Moreover, in several patients with newly diagnosed CD and suffering simultaneously from liver failure due to AILD, GFD was able to reverse hepatic dysfunction, even in cases where liver transplantation was being considered [58,60]. The mechanisms underlying the association between CD and AILD are poorly understood. The most important feature is likely a shared genetic predisposition to autoimmunity [68,69]. Like other autoimmune diseases, CD is a polygenic disorder. In CD, genetic susceptibility is largely associated with specific human leucocyte antigen (HLA) class II molecules on antigen-presenting cells. About 90% of CD patients carry a particular HLA variant (HLA-DQ2.5) that encodes the DQ2.5 molecule, the rest carry HLA-DQ8 or HLA-DQ2.2 [73]. The main genetic marker of CD, HLA-DQ2.5, has a strong linkage disequilibrium with HLA-DR4, HLA-DR52, and HLA-DR3, being the major HLA risk factor for autoimmune liver disease (AIH, PSC, PBC) [74]. Additionally, gene polymorphisms outside the HLA contribute to the genetic susceptibility to both CD and AILD. There are 39 well-established non-HLA loci, including a greater number of independent genetic variants that are associated with CD risk [73]. Many of these loci are related to immune regulations, particularly to B cell and T cell function, and are also shared with other autoimmune diseases, including AILD. The sharing of genetic loci between CD and AILD suggests that there are also shared dysregulated immune responses contributing to the relationship between the two diseases. Moreover, epigenetics, microbiota, and intestinal barrier dysfunction are emerging as potent factors modulating genetic susceptibility and affecting disease manifestation [32,73].

It remains an open question, whether recently described gluten/wheat-related disorders, such as non-celiac gluten sensitivity and wheat-sensitive irritable bowel syndrome have any link to liver diseases [75].

### 6.3. Non-Alcoholic Fatty Liver Disease and Celiac Disease

NAFLD is currently a major cause of chronic liver disease with an estimated global prevalence of approximately 25% [76]. Its occurrence in CD patients is likely a coincidence rather than a true relationship, due to the high frequency of both diseases in the general population [51]. 

Patients with NAFLD are at increased risk for a later diagnosis of CD [77]. The reported prevalence of CD in patients with NAFLD is 2–14% [78]. Conversely, individuals with CD seem to be at increased risk (4–6-times) of NAFLD compared to the general population [52,77]. The relative risk of NAFLD development after a CD diagnosis is higher in the first five years (probably because of excessive weight gain on a GFD) but remained statistically significant even 15 years after a CD diagnosis [77]. GFD could be connected with some dietetic imbalances (such as slightly increased calories, lipid and cholesterol intake, or decreased fiber intake) [79], so patients on GFD are prone to develop NAFLD.

Besides lipotoxicity of specific lipid classes that are stored in hepatocytes, the pathogenesis for NAFLD/NASH may also involve changes in gut permeability, gut microbiota, and other components of gut–liver axis [80]. It has been demonstrated (using the ^51^Cr-EDTA test, by immunohistochemical analysis of zonula occludens protein-1 expression in duodenal biopsy specimens, by the lactulose/mannitol ratio, and by increased levels of circulating zonulin) that NAFLD patients have increased intestinal permeability [81,82]. Moreover, increased intestinal permeability and increased levels of circulating zonulin correlate with the severity of steatosis [82].

NAFLD frequently occurs in type 2 diabetes mellitus and obesity. However, when metabolic syndrome is absent, NAFLD may be related to concomitant CD. Therefore, in the absence of type 2 diabetes mellitus or obesity, and when other causes of liver disease are excluded, patients with NAFLD should be screened for CD [50]. 

Conversely, NAFLD/NASH in patients with CD may also occur as a metabolic sequel of malabsorption and long-standing malnutrition [51]. Malnutrition generates mitochondrial dysfunction in hepatocytes and a reduction in mitochondrial fatty acid β-oxidation, which subsequently leads to abnormal hepatic fat deposition [83]. A GFD usually leads to resolution of liver steatosis and even steatofibrosis in these CD patients [84]. Beyond the mere improvement in nutritional status, a GFD may contribute to restoration of innate immune liver function via harmonization of the gut–liver axis. Luckily, CD disease presenting with severe malabsorption is now uncommon [85]. 

## 7. Clinical Implications: Hepatic Manifestation of Celiac Disease

Liver tests should be routinely checked in all patients with newly diagnosed CD. Hepatic manifestation of CD (i.e., hypertransaminasemia in active CD) deserves specific clinical attention. When a patient’s history and physical and/or ultrasonographic evaluation shows no signs of concomitant hepatic disorder, there is a higher probability of celiac hepatitis than of associated autoimmune liver disease or other coexisting liver disease. Asymptomatic hypertransaminasemia (<5-time the ULN and an AST/ALT < 1) together with a normal physical and ultrasound examination strongly suggests celiac hepatitis. Instead of initial extensive (and expensive) investigations for other causes of liver injury, a “treat-first then re-evaluate” approach is recommended. Liver enzymes should be re-checked after 6–12 months on a strict GFD. If the elevated transaminases return to normal after gluten exclusion, a diagnosis of celiac hepatitis is confirmed, and only follow-ups are recommended, which should optimally include a physical examination, abdominal ultrasound, and liver tests once a year. Because of the nonspecific nature of the findings, liver biopsies are not useful in celiac hepatitis [49,70].

However, lack of normalization of liver enzymes within one year despite adherence to a GFD increases the probability of another concomitant liver disease (of autoimmune, viral, metabolic, or toxic origin). In such cases, a thorough hepatologic examination is necessary [49,70].

Furthermore, patients with elevated liver tests at the time of a CD diagnosis require an extended search for other liver disease, i.e., when the clinical evaluation is not consistent with celiac hepatitis, such as: (1) elevated transaminases levels >5-time the ULN and/or AST/ALT > 1, (2) laboratory signs of cholestasis (i.e., elevated gamma glutamyl transferase, alkaline phosphatase of liver origin, and/or bilirubin), (3) symptomatic liver disorder and/or (4) physical and ultrasonographic signs suggesting a liver disorder. Additionally, abnormal liver tests during follow-up of CD patients who are strictly adhering to a GFD should raise suspicion of other liver disease [49].

## 8. Clinical Implications: Screening of Celiac Disease in Liver Diseases

Screening for CD is recommended in groups of patients with associated AILD (AIH, PBC, PSC), as well as in patients with chronic unexplained hypertransaminasemia, hepatitis or cirrhosis of unknown etiology, and in patients with NAFLD (particularly in those without metabolic syndrome) [8,44,50,58,63,68].

Although the magnitude of the benefit from gluten avoidance relative to reversing autoimmune liver disease in CD patients is controversial, a theoretical context (balance in gut–liver axis and subsequent decline in liver innate immune response) and several of the above-mentioned clinical observations suggest the importance of a GFD. Early detection and treatment of CD may mitigate progression to end-stage liver disease and liver failure [8,58,69].

The first steps in CD screening should include serological testing of TTG2 IgA, together with total serum IgA, followed by subsequent endomysial antibody (EMA) testing in questionable cases (i.e., low titers, possibly false-positive result of TTG2 IgA, which can occur in other autoimmune diseases, etc.). EMA has very high specificity (>99%) for CD. Serologic positivity needs to be confirm with a distal duodenal biopsy [86]. Screening for CD in the context of liver disease deserves special attention in terms of the interpretation of celiac serologic tests, since their accuracy is significantly lower than in individuals without liver disease; especially the guinea pig based anti-TTG2 ELISA testing, which has been shown to have higher rate of false positive results in patients with liver fibrosis. [49]. Thus, in patients with chronic liver disease, EMA evaluation is an important test that can increase diagnostic accuracy. Moreover, immunosuppression (in AILD or after liver transplantation) in the absence of gluten withdrawal can normalize both EMA and TTG2 antibodies. Therefore, in AILD or in end-stage liver cirrhosis, screening for CD must be performed before the start of immunosuppressive therapy or before liver transplantation [49,50]. 

## 9. Conclusions and Recommendation

The liver is a highly immunologically active organ that must simultaneously induce and maintain tolerance while being able to trigger immune responses leading to both protective immunity as well as liver inflammation and fibrosis. An impaired intestinal barrier, dysbiosis, and translocation of bacterial antigens are characteristics of both CD and chronic liver diseases. Disturbances in the gut–liver axis, inhibition of liver TTG2 enzymatic activity, and vitamin D deficiency probably all contribute to liver injury associated with CD. The intestinal microbiome influences the composition of nutrients delivered to the liver and affects metabolic liver functions connected with inflammation. 

A simultaneous clinical presentation of CD with a wide spectrum of liver disorders is well-documented and common. In celiac hepatitis, gluten intake drives liver injury, and a gluten-free diet is the only widely used treatment. Patients with AILD associated with CD can (in addition to specific immunosuppressive treatment) also benefit from a GFD. Overlooked CD can cause chronic hepatitis and rarely liver cirrhosis. Screening for CD should be integrated into the diagnostic routine not only in AIH, PSC, and PBD, but also in unexplained hypertransaminasaemia, chronic hepatitis or cirrhosis of unknown origin, and NAFLD presenting without metabolic syndrome. 

A better understanding of the link between gluten-related immunity and liver injury could contribute to prevention and offer new approaches to the treatment of liver diseases of different etiologies. 

## Figures and Tables

**Table 1:** A role of the components of gut–liver axis in putative pathogenesis of liver injury in celiac disease.

Components of Gut-Liver Axis	Initial Pathogenetic Mechanism	Pathogenetic Consequences in the Intestine	Impact on Liver Immune Cells and Receptors	Consequent Liver Pathology and Altered Liver Physiology
Intestinal barrier	dysregulated intestinal immunity	stimulation of GALT, and entry of intestinal inflammatory cells, cytokines, chemokines, eicosanoids to the liver via the portal vein	triggering immune response via the interaction with liver resident immune cells	liver inflammation and injury
	increased intestinal permeability (e.g., up-regulation of chemokine CXCR3)	increased entry of - food antigens - bacterial antigens (LPS, etc.) - bacterial metabolites (SCFAs, etc.) to the liver via the portal vein	triggering immune response via: - CXCR3 up-regulation - via activation PRRs (CD14/TLR-4 complex, inflammasome, etc.)	liver inflammation and injury altered metabolic regulation (nutrient storage)
Microbiota	dysbiosis	increase of intestinal permeability and inflammation		
		proteolytic activity that can modify the immunogenicity of gliadin peptides		
		enhancement of celiac-disease-associated immunopathology		
		altered bile acid signatures	impact on liver bile salt receptors (FXR, TGR5) and receptor FGFR4	liver and biliary inflammation altered regulation of hepatic bile acids metabolism, and hepatic triglyceride, glucose, and energy homeostasis
Bile	altered bile acid signatures	changes in microbiota composition (dysbiosis)		
	secretion of IgA	changes in microbiota composition (dysbiosis)		
Aberrant lymphocyte homing from the intestine to the liver				liver and biliary inflammation and injury

Abbreviations: FGFR4, fibroblast growth factor receptor 4; FXR, farnesoid X receptor; GALT, gut–associated lymphatic tissue; LPS, lipopolysaccharide; PRRs, pattern-recognition receptors; SCFAs, short chain fatty acids; TGR5, Takeda G-protein receptor 5; TLR-4, toll like receptor 4.

## Abbreviations

AIH	autoimmune hepatitis
AILD	autoimmune liver disease
APCs	antigen presenting cells
CD	celiac disease
DAMPs	damage-associated molecular patterns
DCs	dendritic cells
DNA	deoxyribonucleic acid
FGFR4	fibroblast growth factor receptor 4
FXR	farnesoid X receptor
GALT	gut–associated lymphatic tissue
GFD	gluten free diet
GIT	gastrointestinal tract
HLA	human leucocyte antigen
IFN-γ	interferon γ
IL	interleukin
LPS	lipopolysacharide
MAMPs	microbial-associated molecular patterns
MDSC	myeloid-derived suppressor cells
MHC	major histocompatibility complex
NAFLD	non-alcoholic fatty liver disease
NASH	non-alcoholic steatohepatitis
NK cells	natural killer cells
NKT cells	natural killer T cells NKT cells
NLRs	NOD like receptors
PBC	primary biliary cholantitis
PRRs	pattern-recognition receptors
PSC	primary sclerosing cholangitis
SCFAs	short chain fatty acids
T_reg_ cells	regulatory T cells
TGF-β	transforming growth factor β
TGR5	Takeda G-protein receptor 5
Th cells	T helper cells
TJs	tight junctions
TLRs	toll like receptors
TNF	tumor necrosis factor
TTG2	tissue transglutaminase 2
ULN	upper limit of normal
